# Transcriptome changes reveal the genetic mechanisms of the reproductive plasticity of workers in lower termites

**DOI:** 10.1186/s12864-019-6037-y

**Published:** 2019-09-09

**Authors:** Chenxu Ye, Humaira Rasheed, Yuehua Ran, Xiaojuan Yang, Lianxi Xing, Xiaohong Su

**Affiliations:** 1 Key Laboratory of Resource Biology and Biotechnology in Western China, Ministry of Education, Northwest University, Xi’an, China; 2 College of Life Sciences, Northwest University, Xi’an, China

**Keywords:** Comparative transcriptomes, Gen espression profiles, Reproductive plasticity, Isolated workers, Signal transduction

## Abstract

**Background:**

The reproductive plasticity of termite workers provides colonies with tremendous flexibility to respond to environmental changes, which is the basis for evolutionary and ecological success. Although it is known that all colony members share the same genetic background and that differences in castes are caused by differences in gene expression, the pattern of the specific expression of genes involved in the differentiation of workers into reproductives remains unclear. In this study, the isolated workers of *Reticulitermes labralis* developed into reproductives, and then comparative transcriptomes were used for the first time to reveal the molecular mechanisms underlying the reproductive plasticity of workers.

**Results:**

We identified 38,070 differentially expressed genes and found a pattern of gene expression involved in the differentiation of the workers into reproductives. 12, 543 genes were specifically upregulated in the isolated workers. Twenty-five signal transduction pathways classified into environmental information processing were related to the differentiation of workers into reproductives. Ras functions as a signalling switch regulates the reproductive plasticity of workers. The catalase gene which is related to longevity was up-regulated in reproductives.

**Conclusion:**

We demonstrate that workers leaving the natal colony can induce the expression of stage-specific genes in the workers, which leads to the differentiation of workers into reproductives and suggests that the signal transduction along the Ras-MAPK pathway crucially controls the reproductive plasticity of the workers. This study also provides an important model for revealing the molecular mechanism of longevity changes.

**Electronic supplementary material:**

The online version of this article (10.1186/s12864-019-6037-y) contains supplementary material, which is available to authorized users.

## Background

Termites are social cockroaches, a monophyletic clade nested within the Blattodea, that evolved eusociality more than 150 million years ago [1–3]. Termites live in complex societies that are characterized by a caste system in which few individuals (reproductives including a queen and a king) reproduce, while the large majority (non-reproductives, including workers and soldiers) perform tasks such as foraging, brooding and defence. The reproductive division of labour, with overlapping generations and cooperative brood care, is one of the major evolutionary transitions in termite biology [4, 5], which leads to the rapid development of termite colonies. Recent findings demonstrate that termite nests can increase the robustness of dryland ecosystems to climatic change [6].

**Fig. 1:**
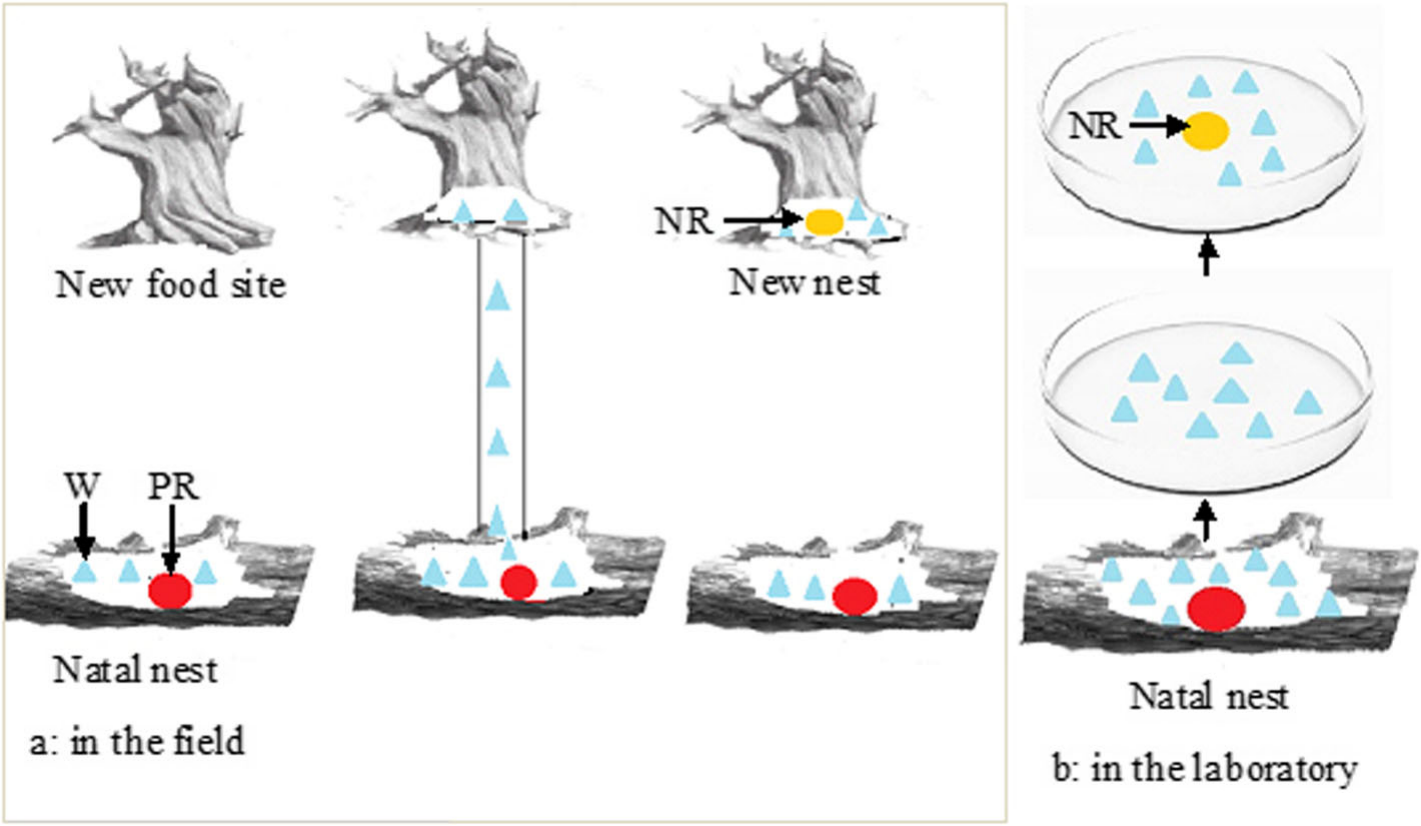
The workers of *Reticulitermes labralis* created new nests (colonies) in the field and laboratory. **a** In the field, the workers created a new colony in new food site (dead wood). The workers exploited new food resources outside the natal nest and moved to a new nest site. The differentiation of female workers into NRs was triggered in the new nest site. **b** In the laboratory, A group of workers from their natal colony was isolated in a Petri dish with moist sawdust. After 4 weeks, the NRs appeared in the group of isolated workers, indicating that a new colony was created. W (blue), workers; PR (red), primary reproductives; NR (yellow), female neotenic reproductives

Lower termites are characterized by a unique flexibility in development. The worker’s potential to differentiate into neotenic reproductives (replacement reproductives) remains one of the great mysteries of termite biology [4]. The diverse and flexible breeding systems found in termites pre-adapt them to invade new or marginal habitats [7]. In the genus *Reticulitermes*, caste differentiation bifurcates into two pathways: the nymphal (sexual imaginal) pathway, which develops into reproductives (alate adults as primary reproductives and brachypterous neotenic reproductives), and the apterous pathway, which leads to workers and soldiers. Reproductives and soldiers die without workers’ help, and all soldiers are sterile. However, workers have unique flexibility in that a worker has the capability to develop into apterous neotenic reproductives that develop in the absence of reproductives to provide for continued growth of the colony [4, 8, 9]. The female workers are not able to produce mature eggs unless they moult into conspicuous reproductive females, whereas the male workers can copulate with queens [10–12]. Strikingly, workers can exploit new food resources outside the natal nest and will move to a new nest site when food supplies are low, and then the reproductive development of the female worker can be triggered in the new nest site (Fig. 1). The reproductive plasticity of workers provides colonies with tremendous flexibility to respond to environmental changes and the deterioration of the nest and food resources. Therefore, this caste system of termites is considered to be the basis for their evolutionary and ecological success [4].

All members in a termite colony have the same genetic background. Differences in the complex traits among castes are not the result of the presence or absence of particular genes but arise from changes in the mechanisms of gene regulation affecting when and where a gene or an entire regulatory module is expressed [4, 13]. The transition of workers into reproductives is caused by specific gene expression that depends on environmental and social stimuli. Current evidence demonstrates that chemical communication by a queen prevents the development of worker reproduction [14]. The worker caste is seen as a neuter caste whose sexual development is suppressed due to counterbalanced maternal and paternal imprinting [15]. A group of above 12 isolated workers from their natal colony of *Reticulitermes* can produce a female reproductive after 4 weeks, and then a new colony is founded [9, 11, 16]. In termite caste differentiation, the differentiation of the workers into reproductives may be the most difficult process [11], in which the specific expression of genes is induced. However, this specific expression profile remains unclear.

In the present study, to gain insights into the molecular mechanisms underlying the reproductive plasticity of workers, we induced the female workers that transformed into reproductives by using the groups of isolated workers of *R. labralis*, and then sequenced the transcriptomes of individuals at three stages: female workers in natal colonies in which there were queens, isolated female workers from their natal colonies (IWs) and female neotenic reproductives (NRs) differentiated from isolated workers. First, we identified transcripts undergoing significant abundance changes during worker development into reproductives and identified 38,070 differentially expressed genes (DEGs, including upregulated and downregulated genes). Second, we found a pattern of gene expression involved in the differentiation of workers into reproductives by a trend analysis of the DEGs enrichment. Third, 25 signal transduction pathways classified into environmental information processing were involved in the differentiation of workers into NRs in the absence of queens, and then we performed qRT-PCR analysis of five Ras signalling pathway-related genes. Lastly, the differential expression of the Catalase gene related to longevity were found in the female workers, isolated workers and NRs. This is the first comprehensive study on the gene expression of differentiation of workers into reproductives.

## Results

### Morphological changes of the workers developing into neotenic reproductives

**Fig. 2:**
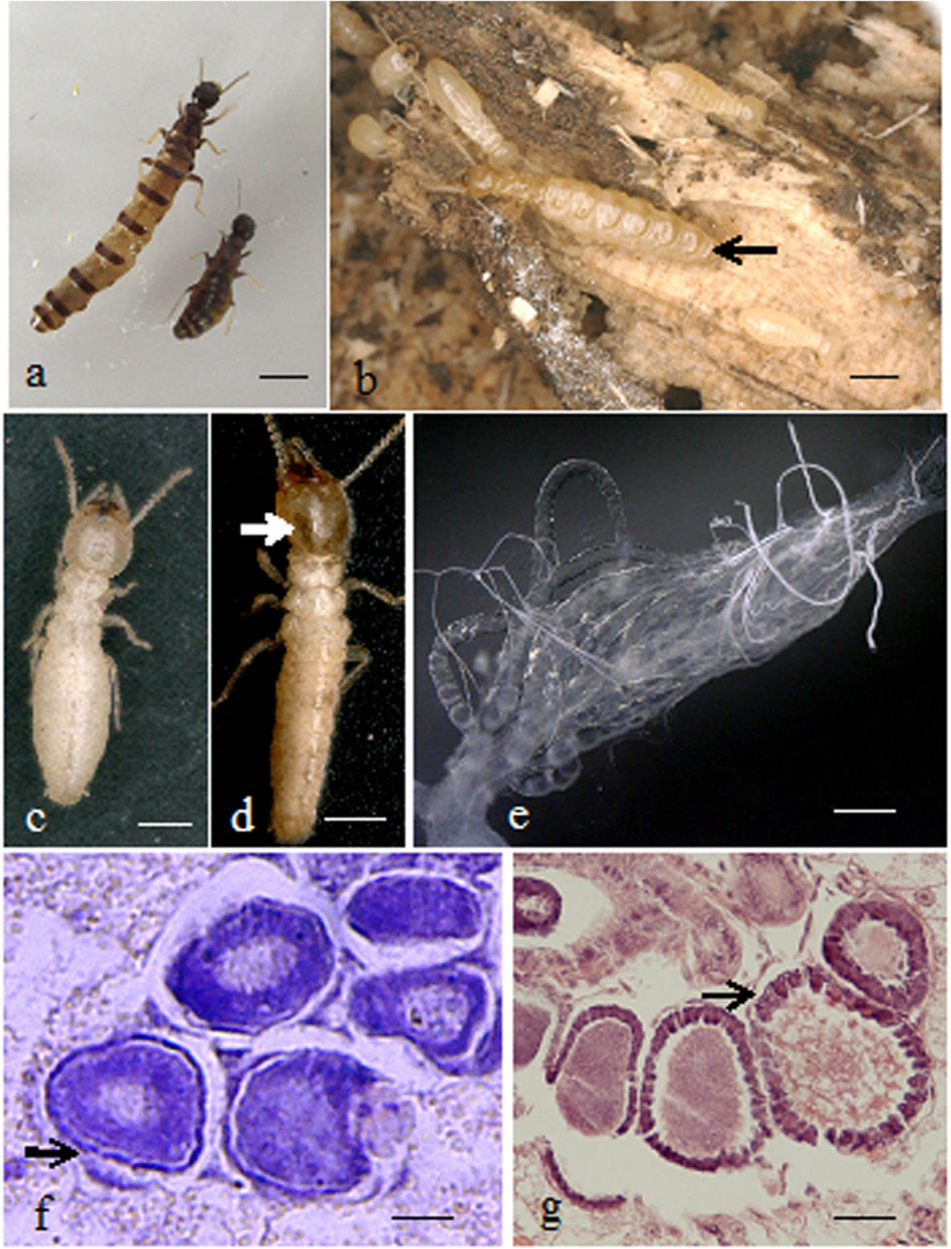
Morphological changes of the workers developing into neotenic reproductives (NRs). **a** The primary reproductives with darkened pigmentation. Scale bar =1.0 mm. **b** Neotenic reproductives in colony. Scale bar =1.0 mm. **c** A female workers. Scale bar =1.0 mm. **d** The neotenic reproductives derived from female workers had brown pigmentation on their heads. Scale bar =1.0 mm. **e** The ovary of neotenic reproductives. Scale bar = 100 μm. **f** Each oocyte in workers was surrounded by degenerated follicle cells. Scale bar = 20 μm. **g** Each oocyte in the neotenic reproductives was surrounded by a thick layer of cube-shaped follicle cells. Scale bar = 25 μm

The differentiation of neotenic reproductives (NRs) was induced by isolating late instar workers of *R. labralis* (Fig. 1). The difference in morphology between the female workers and NRs was significant (Fig. 2a-d). The abdomen lengths of the workers, isolated workers and NRs were 1.89 ± 0.06,1.95 ± 0.03 and 2.70 ± 0.11 mm, respectively. The abdomen length significantly increased after the female workers moulted into NRs. The heads of the female NRs had brown pigmentation stripes. No male NRs emerged in experimental colonies. Moreover, the ovaries of the workers produced degenerate follicles [12]. In the ovaries of the workers, a conspicuous feature was that each oocyte was surrounded by a thin layer of follicle cells in which the elongated follicle cells were degenerated (Fig. 2f). In NRs, each oocyte was surrounded by a thicker layer of follicle cells (Fig. 2e and g).

### RNA sequencing and de novo transcriptome assembly

A total of 460,864,858 clean-sequence reads were obtained by transcriptome sequencing (Additional file 1). Based on the clean reads, 112,954 unigenes ranging from 201 bp to 38,952 bp were assembled. The mean length was 848 bp, and the N50 length was 1536 bp (Additional file 2 and Additional file 3).

### Function annotation

**Fig. 3:**
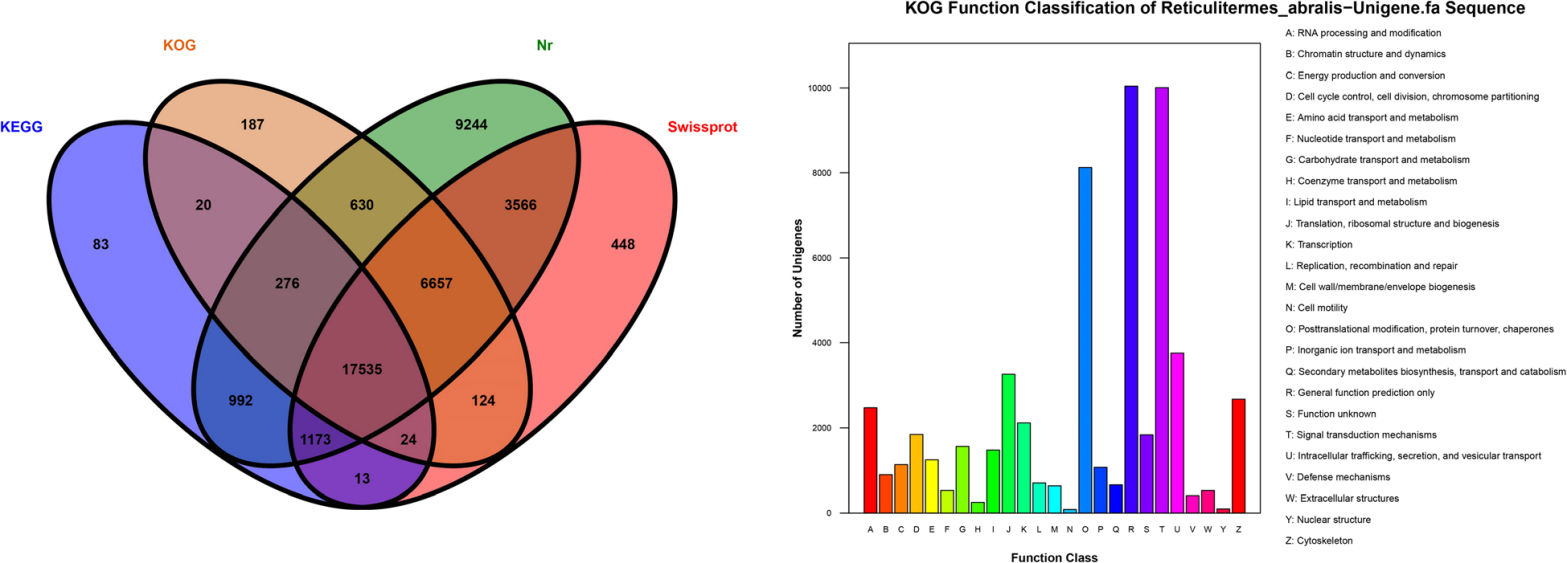
A total of 112,954 unigenes were used for functional annotation. **a** Venn diagram of the distribution of unigene and database matching results. The numbers of unique sequence-based annotations is the sum of the unique best BLASTX hits from the Nr, Swiss-Prot, KOG and KEGG databases. The overlapping regions between the four circles contain the numbers of unigenes that shared BLASTX similarities with the respectively databases. **b** Histogram presentation of the clusters of the KOG function classifications. A total of 25,453 unigenes were grouped into 24 KOG classifications. The y-axis indicates the number of unigenes in a specific functional cluster. The legend presents the 24 functional categories

We annotated 112,954 unigenes. The Venn diagram illustrates that the number of unique sequence-based annotations is the sum of the best unique BLASTx hits from the Nr, Swiss-Prot, KEGG and KOG databases (Fig. 3). A total of 40,972 unigenes (36.27%) were successfully annotated to at least one database. 17,535 unigenes (15.52%) were successfully annotated in the four databases. In total, 40,073 (35.48%) unigenes had significant matches in the Nr database, followed by 29,540 unigenes (26.15%) in the Swiss-Prot database. 25,453 (27.99%) and 20,116 unigenes (22.53%) had specific matches in the KOG and KEGG databases, respectively (Fig. 2a and Additional file 4). As expected, the top hits were found in the insect genomes, especially termite *Zootermopsis nevadensis* (25.82%) (Additional file 5).

Based on the Nr protein database annotation, we classified the function of the predicted unigenes using GO, KOG and KEGG (Fig. 3). In the GO functional classification, the annotated unigenes belonged to clusters of biological process (30,717 unigenes, 36.68%), molecular function (29,711 unigenes, 35.48%) and cellular components (23,312 unigenes, 27.84%), which were distributed into 55 categories (Additional file 6). For the KOG database, 25,453 unigenes were classified into 25 functional categories (Fig. 3). The largest group was general function prediction(10,042 unigenes, 39.45%), followed by signal transduction mechanisms (10,007 unigenes, 39.31%), posttranslational modification, protein turnover, and chaperones (8124 unigenes, 31.92%). A total of 20,116 unigenes were annotated into 233 pathways in the KEGG database. The most enriched pathway was “Ribosome” (1533 unigenes, 6.26%), followed by “Protein processing in the endoplasmic reticulum” and “Endocytosis”, for which the unigene percentages were 4.48 and 3.36%, respectively (Additional file 7).

### Differentially expressed genes (DEGs) in workers, isolated workers and neotenic reproductives

**Fig. 4:**
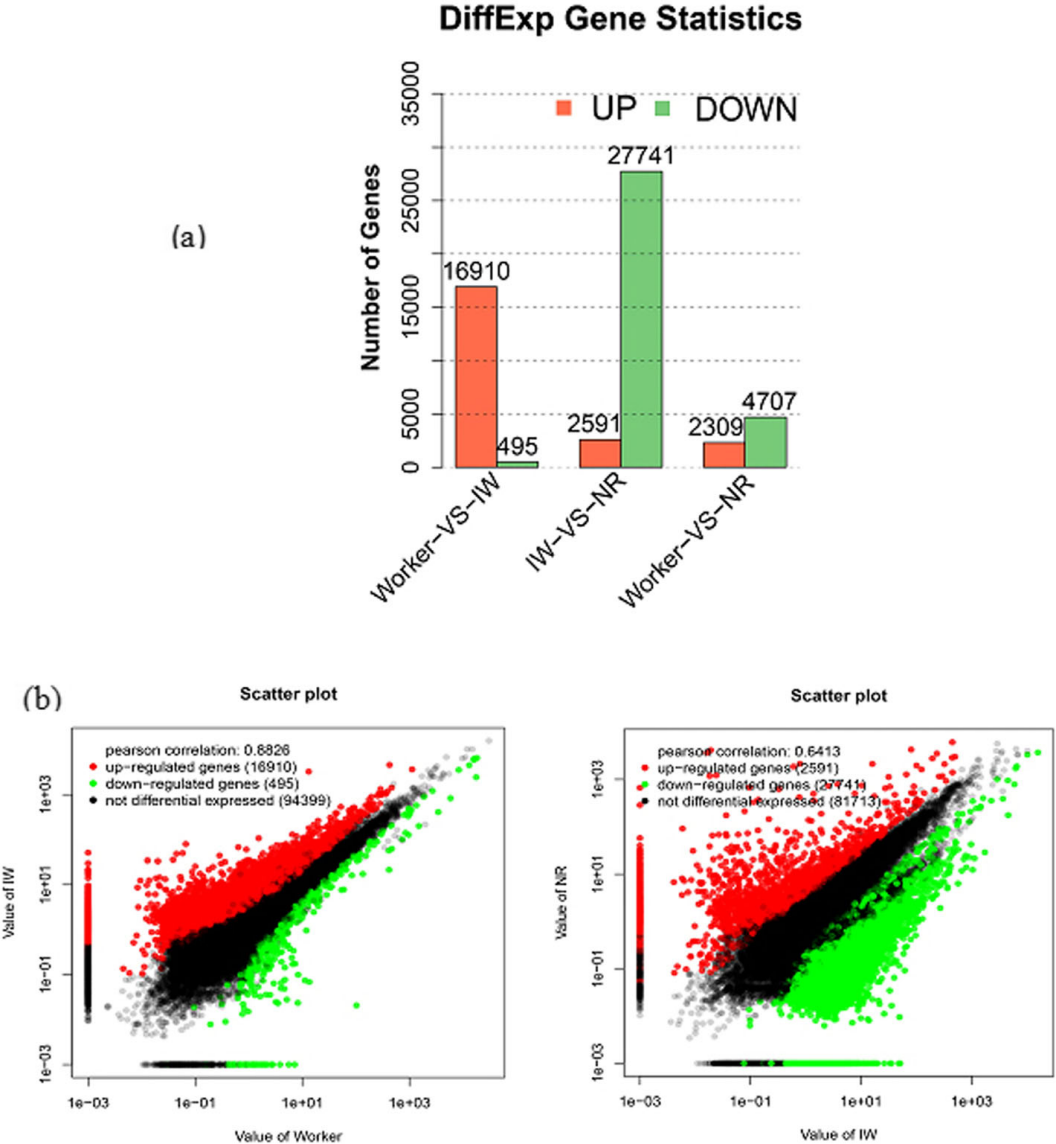
Differentially expressed genes in workers, IWs and NRs. **a** The number of the differentially expressed genes in workers, IWs and NRs. The x-axis indicates the three stages (worker: female workers in natal colonies; IW: isolated female workers from their natal colonies; NR: female neotenic reproductives differentiated from isolated workers). Red represent transcripts that were significant up-regulated, and green indicate that those transcripts were significantly downregulated. The parameters FDR ≤ 0.001 and log2Ratio ≥ 1 were used as the thresholds to judge the significance of gene expression differences. **b** The scatter plot of the differentially expressed genes in workers, IWs and NRs. Red and green scatter plot represent upregulated genes and downregulated, respectively. Black scatter plot represent that the genes were not differential expressed. IW, isolated workers; NR, neotenic reproductives

We performed a quantitative comparison of the gene expression between workers and IWs (worker vs IW), between IWs and NRs (IW vs NR), and between workers and NRs (worker vs NR) (Fig. 4). We identified 38,070 DEGs (upregulated and downregulated genes) among workers, IWs and NRs. There were 17,405 DEGs in “workers vs IWs”, 30,332 DEGs in “IWs vs NRs” and 7016 DEGs in “workers vs NRs”. The results showed that “worker vs NR” had the fewest number of DEGs compared with “worker vs IW” and “IW vs NR”. The number of DEGs from “IW vs NR” and “worker vs IW” was 2.5-fold and 4.3-fold, respectively, compared with the number of DEGs of “worker vs NR”. Of the 17,405 DEGs, 16,910 (97.2%) genes were upregulated in IWs compared with workers. After the IWs developed into NRs, most of the DEGs (27,741, 91.5%) were downregulated.

### Trend analysis of DEGs enrichment across the developmental stages of workers, IW into NRs

**Fig. 5:**
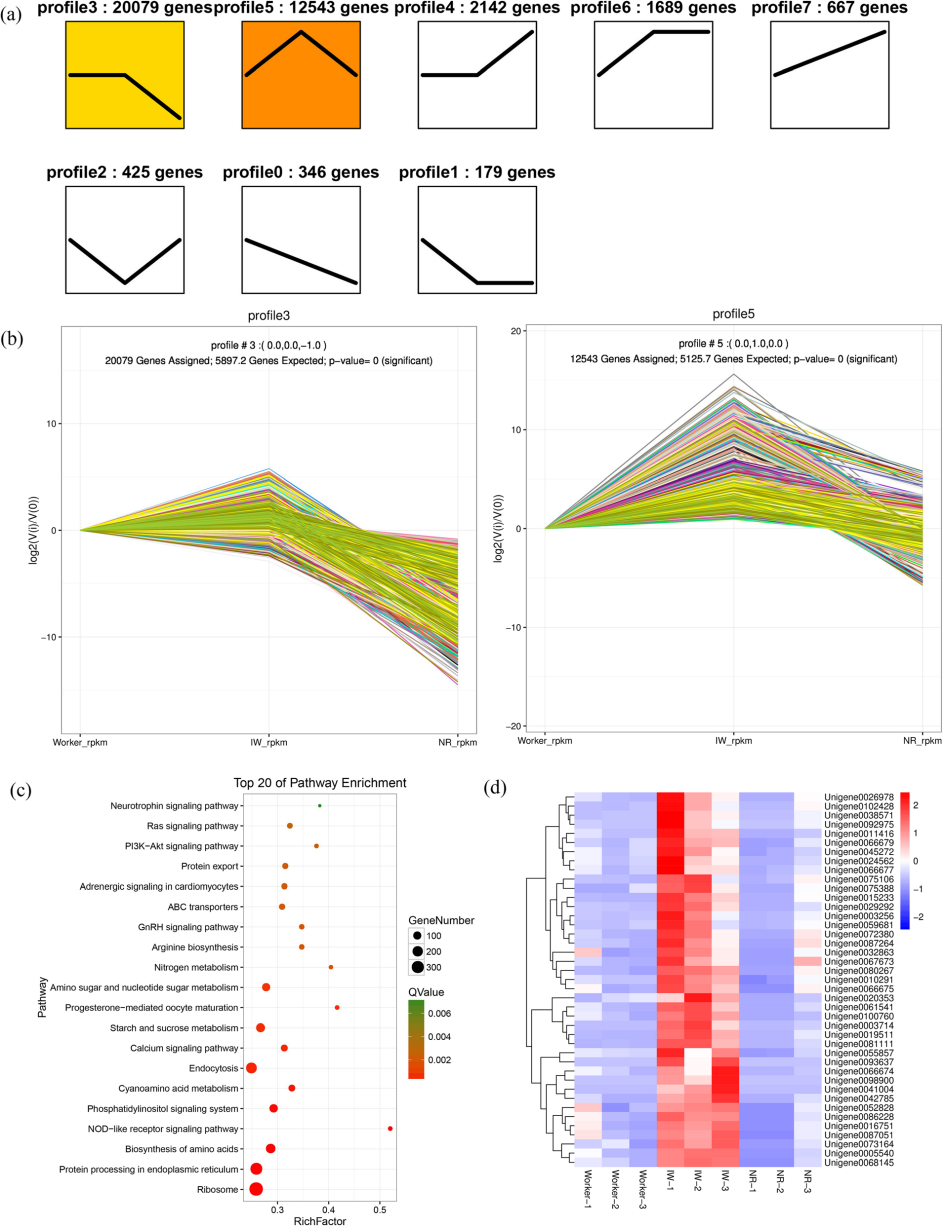
Trend analysis of DEGs enrichment across the developmental stages of workers, IW into NRs. **a** Trend analysis of differentially expressed genes. The 38,070 DEGs from workers, IWs and NRs of *R. labralis* were clustered into 8 profiles. **b** Significantly enriched trend analysis of profile 3 and profile 5. **c** Thop 20 of KEGG pathway enrichment of DEGs in Profile 5.The bubble size represents the number of DEGs, and the bubble color represents the Q value. **d** Heat map of the DEGs for the 41genes involved in Ras signaling pathway. IW, isolated workers; NR, neotenic reproductives

The 38,070 DEGs from workers, IWs and NRs of *R. labralis* were clustered into 8 profiles (Fig. 5a), in which 32,622 (85.69%) genes were in profile 3 and profile 5 (Fig. 5b). Only in profile 5, all 12,543 genes were specifically upregulated in IWs and were downregulated in workers and in NRs. The expression levels of the genes in profile 5 were significantly higher in IWs than those in the workers and NRs, which indicated that these genes were specifically overexpressed during the stage of IWs developing into NRs, and then were rapidly downregulated after the IWs became NRs. The genes in profile 5 were involved in the differentiation of IWs into NRs. Therefore, we focused on the gene expression in profile 5.

To determine the biological function of the DEGs in profile 5, the GO classification and the KEGG pathway analysis were carried out. All of the DEGs were annotated in the GO database and were categorized into 51 functional groups, including the three main GO ontologies: biological process, cellular component and molecular function (Additional file 8). Of these DEGs, a large number were dominant in catalytic activity, binding, metabolic processes, cellular processes, the cell part and the cell.

Our KEGG pathways analysis showed that 2272 DEGs in profile 5 were mapped in 201 pathways, in which 31pathways were significantly related to IWs differentiation into NRs (Q-value < 0.05), including ribosomes, protein processing, biosynthesis of amino acids, calcium signalling pathway, etc. (Additional file 9). Five **s**ignal transduction pathways were involved in profile 5, and the signal transduction pathways in the top 20 pathways were the phosphatidylinositol signalling pathway, the calcium signalling pathway, the Ras signalling pathway and the PI3K-Akt signalling pathway (Fig. 5c). The number of genes enriched in the above pathways were 111 (4.89%), 67 (2.95%), 41 (1.8%) and 27 (1.19%) unigenes, respectively. All of the five signal transduction pathways in profile 5 were classified into environmental information processing, which indicated that these signal transduction pathways were related to the transition of workers into NRs after the workers were isolated from their natal colonies.

Active transposable elements (TEs) can “jump” within the genome, thereby disturbing the regulation and expression of other genes by transposing into another gene. In profile 5, 2 of 739 annotated TE genes were upregulated in IWs, one PogoTE gene and one PiggyBac TE gene, and then were downregulated in NRs.

### Signal transduction along the Ras-MAPK pathway axis from plasma membrane to nucleus

Ras proteins transmit signals from cell surface receptors to a variety of effectors and thereby regulate pathways that govern cell proliferation and differentiation. Ras proteins are binary switches that cycle between ON and OFF states during signal transduction [17]. In profile 5, we found that 41 genes involved in the Ras signalling pathways were significantly upregulated in IWs compared with workers, and then were significantly downregulated in NRs (Fig. 5d). All expression levels of genes in the IWs were significantly higher than those in the workers (natal colonies) and in the NRs.

**Fig. 6:**
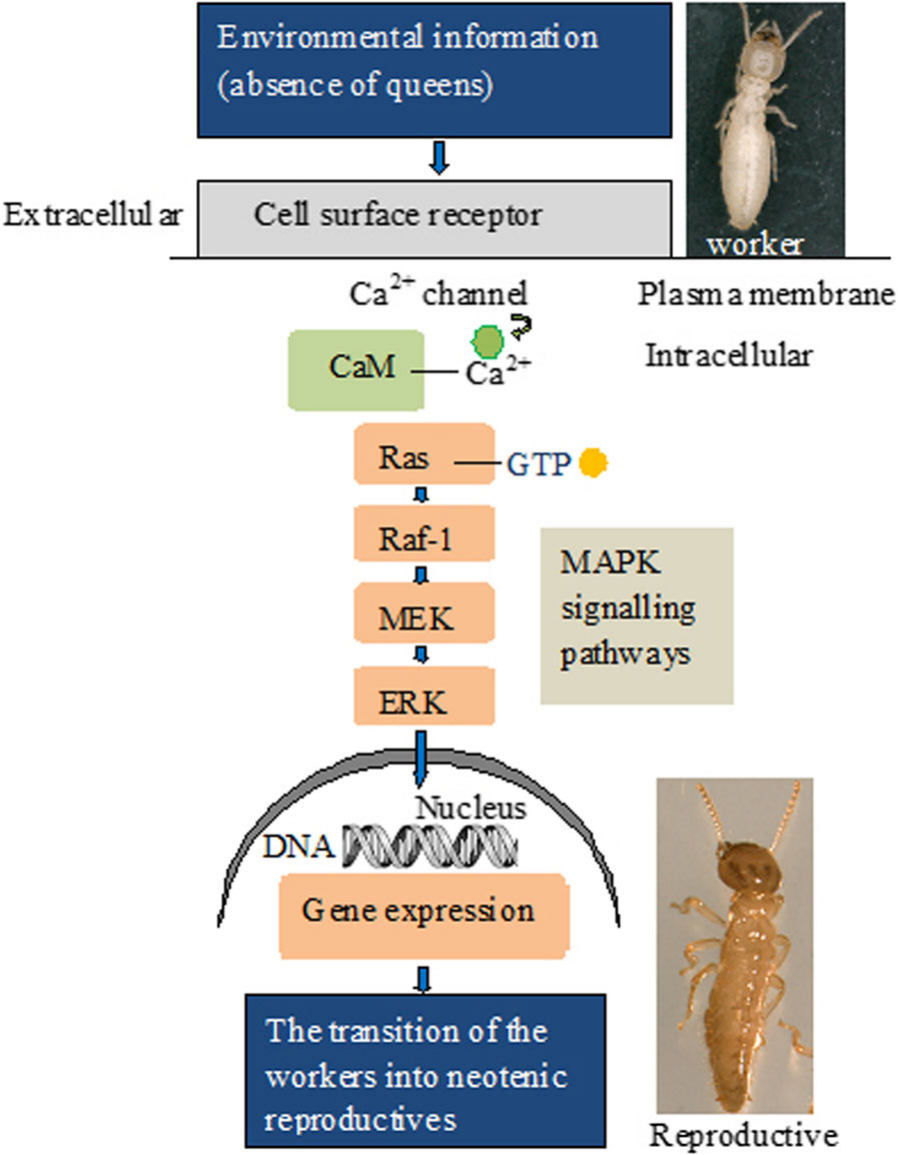
Predicted model for Ras-MAPK pathway controlling the differentiation of workers into neotenic reproductives. When cell surface receptor accepts extracellular signaling from the absence of queen pheromone, a increase in intracellular Ca^2+^ is triggered via Ca^2+^ channels. Ca^2+^ enters the cytoplasm where it binds and activates calmodulin (CaM). Ca^2+^-CaM can act on Ras in the cytoplasmic surface. Ras binds GTP. Activated Ras initiates the Raf-1, and through the MEK/ERK MAPK pathway, triggers signaling to the nucleus

The transcriptome analysis showed that a Ras (N-Ras protein) gene was expressed only in IWs and was not detected in workers and NRs, and Ras-extracellular signal regulated kinase (ERK) was significantly upregulated in IWs. The signalling pathways in profile 5 showed that Ca^2+^- Calmodulin (Ca^2+^-CaM) involved the activation of the Ras signalling pathway, and then activated the downstream effector pathway MAPK during IW development into NRs (Additional file 10). We identified that 52 genes in the MAPK signalling pathways was significantly upregulated in IWs and was significantly downregulated in workers and in NRs. 61 genes involved in the calcium signalling pathway were significantly upregulated in IWs and were significantly downregulated in workers and in NRs. Predicted model for Ras-MAPK pathway controlling the differentiation of workers into neotenic reproductives (queens) was shown (Fig. 6).

### Dynamic changes in Ras signalling pathway-related gene expression during the differentiation of workers into NRs

**Fig. 7:**
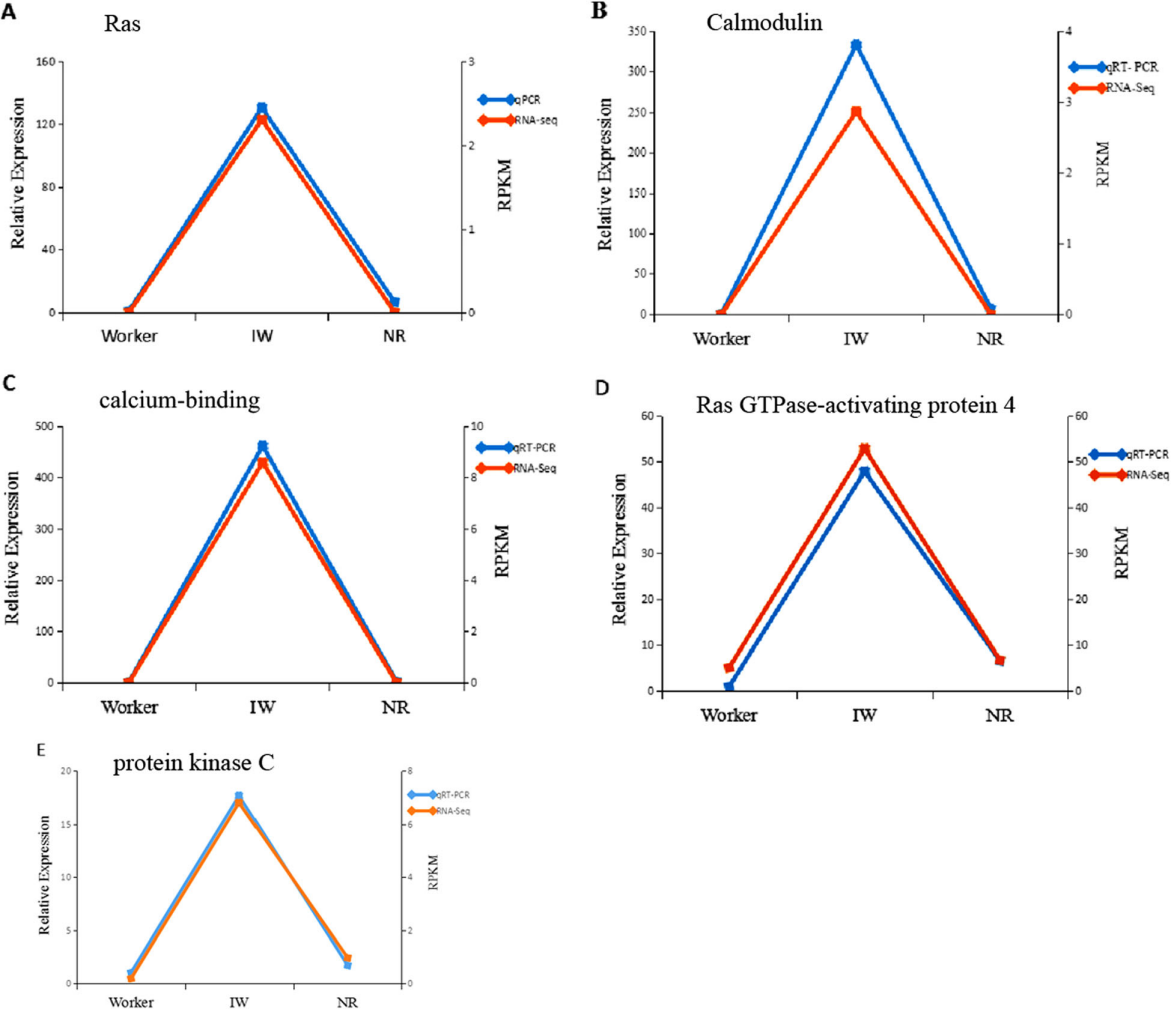
The Comparison of expression of five genes between RNA-Seq and qRT-PCR during the differentiation of workers into NRs. **a** Ras protein; (**b**) Calmodulin; (**c**) calcium-binding protein; (**d**) Ras GTPase-activating protein 4; (**e**) protein kinase C. Worker, the female workers in natal colonies; IWs, isolated female workers for 3 weeks; NRs, female neotenic reproductives. The blue color represents the qRT-PCR analyses of the gene expression levels. The red color represents the transcriptome sequencing analyses (RPKM) of the gene expression levels

We performed qRT-PCR analysis on the expression of five Ras signalling pathway-related genes. The expression levels of the five genes in the isolated female workers (IWs) were higher compared with the expression levels of the workers and neotenic reproductives (NRs). The relative expression level of Ras in the IWs was 131-fold and 20-fold higher than the expression in the workers and NRs, respectively (Fig. 7a). The relative expression level of Calmodulin (CaM) in the IWs was 333-fold and 47-fold higher than the levels in the workers and NRs, respectively (Fig.7b). The relative expression level of the calcium-binding protein in the IWs was 462-fold and 205-fold higher than the levels in the workers and NRs, respectively (Fig. 7c). The relative expression level of the Ras GTPase-activating protein 4 (Ras-GDP) in the IWs was 48-fold and 7-fold higher than the levels in the workers and NRs, respectively (Fig. 7d). The relative expression level of protein kinase C (PKC) was 18-fold and 11-fold higher in the IWs than the levels in the workers and NRs, respectively (*p* < 0.05) (Fig. 7e). The five genes in the IWs were extremely upregulated. After the IWs differentiated into NRs, the expression levels of the five genes in the NRs derived from IWs extremely decreased. There were no significant differences in the expression levels of the five genes between the workers and the NRs.

The data from transcriptome sequencing also showed that the five genes were significantly upregulated in IWs compared with workers and NRs, and there were no significant differences in the expression levels of the five genes between the workers and the NRs. The expression of Ras, Calmodulin and calcium-binding genes in the workers and NRs was not detected. Our results showed that the expression profiles of the candidate unigenes revealed by the qRT-PCR data were consistent with those derived from transcriptome sequencing (Fig. 7a-e), which indicate that the RNA-Seq analysis was reliable and provided a valuable gene sequence for biological analysis. Ras signalling pathway-related genes exhibited a significant IW-specific overexpression and have putative associations with the reproductive plasticity of workers.

### Dynamic changes in the expression levels of catalase gene related to longevity during the differentiation of workers into NRs

**Fig. 8:**
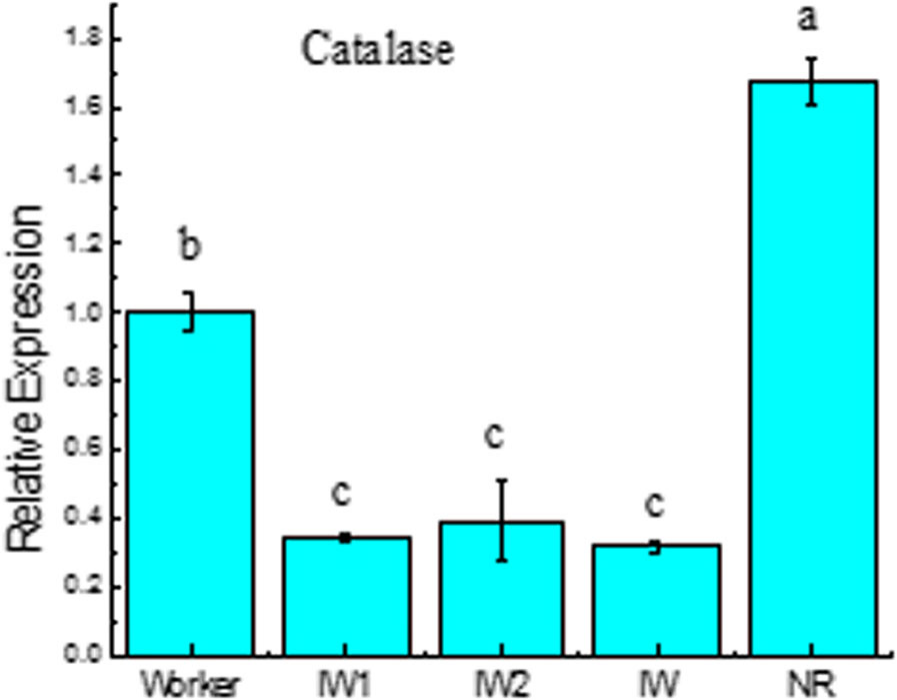
Dynamic changes in the expression levels of catalase gene related to longevity during the differentiation of workers into NRs by qRT-PCR analysis. Error bars represent the standard deviation of the mean. Different letters over the bars denote significant differences at *p* < 0.05. Worker, the female workers in their natal colonies; IW_1_, isolated female workers for 1 week; IW_2_, isolated female workers for 2 weeks; IW, isolated female workers for 3 weeks; NRs, female neotenic reproductives

The oxidative stress theory of ageing states that the accumulation of oxidative damage causes ageing. In the model insect *Drosophila melanogaster*, overexpression of catalase (CAT) resulted in reduced levels of oxidative stress and an extended lifespan, which indicated that CAT positively effects longevity [18, 19]. In this study, the CAT of *R. labralis* closely matched the CAT gene sequence of the termite *Zootermopsis nevadensis*. We found a dynamic change in the expression of the CAT gene during the differentiation of workers into reproductives. Our qRT-PCR analysis showed that the expression levels of the CAT gene decrease significantly in isolated female workers for 1 week (IW_1_), isolated female workers for 2 weeks (IW_2_) and isolated female workers for 3 weeks (IW) compared with those of workers. However, after the IW moulted into NR, the expression levels of the CAT gene increased significantly, and the expression levels of the CAT gene in NRs (1.670 ± 0.065) was approximately 5-fold higher compared with those of IW(0.316 ± 0.012) (Fig. 8). Our KEGG pathway analysis showed that the CAT gene, as a downstream gene in the longevity-regulating pathway, directly caused longevity. However, the expression of CAT was usually suppressed by the Ras-P13k-Akt-FOXO pathway (Additional file 11).

## Discussion

For the first time, the changes of gene expression during the differentiation of workers into neotenic reproductives were revealed using de novo sequencing of stage-specific transcriptomes. All colony members share the same genetic background, and differences in the caste are caused by differences in gene expression [13]. In this study, we identified genes that were specifically overexpressed in isolated female workers (IWs) of *R. labralis*. Of 17,405 DEGs, 16,910 (97.2%) genes were upregulated in IWs compared with that of workers in natal colonies. After isolated workers developed into reproductives, most of the DEGs (27,741, 91.5%) were downregulated. These results confirmed that the isolated workers had the most distinctive gene-expression pattern, with the vast majority of genes were being specifically overexpressed in the absence of queens. Therefore, we suggest that when workers leave the natal colony or a queen dies, the expression of stage-specific genes is induced in workers, which leads to the differentiation of workers to reproductives.

We confirm that profile 5 of the DEGs shows a pattern of gene expression that is involved in the differentiation of the workers to reproductives. In this study, although the 38,070 DEGs from the workers in natal colonies, IWs and NRs of *R. labralis* were clustered into 8 profiles, only the expression of genes in profile 5(12,543 DEGs) were not detected or were very low in workers, and then were specifically upregulated in IWs and downregulated in NRs. Therefore, it is conceivable that the expression of genes in profile 5 in female workers was affected by the presence or absence of queens. The most surprising finding is that, according to the KEGG pathways analysis, five signal transduction pathways in profile 5 were classified into environmental information processing, which indicated that these signal transduction pathways were involved in the transition of workers into reproductives after the workers were isolated from their natal colonies.

Our transcriptome analysis showed that a Ras gene was only expressed in IWs and was not detected in the workers and NRs. Moreover, the qRT-PCR analysis confirmed that the relative expression level of Ras in the IWs was 131-fold and 20-fold higher than that of the workers and NRs, respectively, which indicated that Ras was especially overexpressed in workers in the absence of queens. β-Subunit of the voltage-gated Ca2+ channel Cav1.2 drives signaling to the nucleus via H-Ras [20]. All Ras biology occurs in membranes, and Ras genes act as molecular switches. Ras subfamily members work with certain membrane receptors, such as receptor tyrosine kinases. When these receptors accept extracellular signalling factors, such as hormones and growth factors, signalling cascades start via the phosphorylation of proteins. Ras acts as a switch in the middle of such signalling cascades to regulate downstream signalling pathways [21, 22]. Chemical communication by a reproducing queen prevents worker reproduction in termite colonies [14]. A queen-produced volatile pheromone consisting of n-butyl-n-butyrate and 2-methyl-1-butanol has been identified in *R. speratus*. This queen pheromone inhibits the differentiation of workers into neotenic reproductives [23, 24]. Moreover, the female workers transform into reproductives via moulting. Previous studies have confirmed that the activity of Ras in the *Drosophila* prothoracic gland induces precocious ecdysone release [25]. Obviously, the signal of queen death combined with the receptor in workers can activate Ras on the cell membrane, and then Ras transmits the signal to the downstream pathway, which leads to the specific expression of genes. We suggest that Ras proteins are binary switches that cycle between ON and OFF states during the signal transduction of worker reproductive options.

We infer that Ras stimulated the Ras-ERK MAPK signalling pathway to induce and promote the ovary growth of workers in the absence of queens. Ras is a key component of growth signalling pathways, because it functions as a relay switch between upstream growth receptors and downstream effector pathways, such as MAPK [17]. Gonadal development during the development of the workers into neotenic reproductives appears to be the most significant biological process that leads to the formation of caste-specific differences in tasks and statuses. In the termite *R. speratus*, the average size of the ovaries from all of the emerged female neotenic reproductives in isolated worker groups was significantly larger compared to females that retained their worker status [10]. Our previous study showed that the mean length and width of the ovaries of *R. labralis* increased 2.5-fold and 3-fold longer, respectively, during the differentiation of isolated workers into reproductives [11]. Degeneration of the follicles in the workers was found, particularly, when compared with the nymphs. Degenerate follicle cells that fail to support further development of oocytes is an important reason for the absence of vitellogenesis during oogenesis in the workers, which is a leading cause of reduced fertility in the workers [12]. To survive and propagate, organisms must respond to changes in the environmental condition by altering their physiology and behaviour. Reproductive development is particularly well-tuned to changes in environmental conditions [26]. The germ line utilizes the well-conserved Ras-ERK signalling pathway in different contexts [27, 28]. In *Caenorhabditis elegans*, in the presence of food, the DAF-2 insulin-like receptor signals through the Ras-ERK pathway to drive meiotic prophase 1 progression and oogenesis; in the absence of food, the downregulation of the Ras-ERK pathway stalls oogenesis^25^. We found that, in the absence of queens, Ras and ERK genes had higher expression in workers, and the Ras-ERK signalling pathway axis regulated the gene oocyte meiosis pathway. Thus, we further conclude that “the absence of the queen pheromone” signals through activation of the Ras-ERK signalling pathway to drive the ovary development of isolated workers, and the Ras/MAPK signalling pathway axis regulates gene expression, proliferation and differentiation in the ovary.

We found that as soon as an individual worker moulted into a reproductive, the CAT gene in the longevity regulation pathway was activated immediately, which indicated that longevity is related to the status of the individual termites. The trade-off between reproduction and longevity is known in wide variety of animals. Social insect queens are rare organisms that can achieve a long lifespan without sacrificing fecundity [19]. Reproducing queens in termites can live for 20 years, whereas workers live only a few weeks to months. The queens and workers in a colony share the same genetic background, and differences in longevity are caused by differences in gene expression [13]. In the termite *R. speratus*, queens showed more than seven times higher expression levels of CAT genes than those of workers [19]. This efficient antioxidant system can partly explain why termite queens achieve a long life span [19]. In our study, after IWs molted into NRs, the expression level of the CAT gene was increased significantly, and the expression levels of the CAT gene in NRs were approximately 5-fold higher compared with that of IW. These results suggest that in termites, short-lived individuals (workers) can become long-lived individuals (queens), and the expression of longevity genes is influenced by the colony environment. Isolated workers face enormous environment stress because they need found new nests and resist pathogen and parasites. We found that the expression levels of the CAT gene decrease significantly in isolated workers compared with those of workers in natal colonies. Therefore,we suggest that the lifespan of workers decreases after leaving their natal colonies. This study provides an important model for revealing the molecular mechanism of longevity changes.

## Conclusion

We identified 38,070 differentially expressed genes and found profile 5 to be the pattern of gene expression involved in the differentiation of the workers into reproductives. 12,543 genes were specifically upregulated in the isolated workers. We suggests that the signal transduction along the Ras-MAPK pathway crucially controls the reproductive plasticity of the workers and short-lived individuals can become long-lived individuals by the transition of castes.

## Methods

### Differentiation of isolated workers into neotenic reproductives

Mature *R. labralis* colonies were collected from Daxingshan Temple in Xi’an City, China, in 2015. The nest woods were brought back to the laboratory, were kept in plastic cases (80 × 50 × 40 cm^3^) and were covered with wet soil. To induce the differentiation of female workers into neotenic reproductives, we established 100 groups of isolated workers from their natal colonies in April of 2016. A group of isolated workers contained 50 late instar workers and two soldiers in a Petri dish with moist sawdust. After 4 weeks, the female neotenic reproductives appeared in groups of isolated workers. The neotenic reproductives were distinguished from workers by a long abdomen and dark brown pigmentation. The sex of the individuals was determined by morphological observation of the seventh sternite. We collected the late instar female workers in their natal colonies, isolated late instar female workers for 1 week (IW_1_), isolated late instar female workers for 2 weeks (IW_2_), isolated late instar female workers for 3 weeks (IWs) and female neotenic reproductives (NRs) derived from isolated workers. The abdomen lengths of the workers, isolated workers and neotenic reproductives were expressed as the mean ± SD. The significant differences were identified with the non-parametric Kruskal-Wallis test followed by Dunn’s multiple comparisons test. *P*-values< 0.05 were considered as significant. We removed the abdomens and then froze head-thoraxes in liquid nitrogen for RNA extraction.

### Oocyte development

The fixed workers and NRs were dehydrated in an ascending ethanol series and embedded in paraffin. Longitudinal sections that were 6 mm thick were collected on polylysine-coated slides. The sections were deparaffinised and rehydrated, then stained using haematoxylin and eosin. The sections showed oogenesis of the workers and NRs.

### RNA extraction, cDNA library construction and sequencing

The total RNA of the head-thoraxes of female workers, IWs and NRs (three biological replications) was extracted using RNAiso Plus reagent (TaKaRa Bio. Inc., Japan) according to the manufacturer’s protocol. RNA quality was verified using a Nanodrop spectrophotometer, Qubit 2.0 and an Agilent 2100 Bio-analyser (Agilent Technologies, CA, USA). Next, mRNA was isolated from the total RNA using oligo (dT) magnetic beads (Qiagen Co., Ltd., Shanghai, China); then, the fragmentation buffer was added to the beads coated with mRNA, and the mRNA was broken randomly. These short fragments were used as templates for the random hexamer-primed synthesis of first-strand cDNA. The second-strand cDNA was synthesized using RNase H, dNTPs and DNA polymerase I. Then, the double-stranded cDNA (dsDNA) was further purified using the Qia Quick PCR extraction kit and was resolved with the EB buffer for end reparation and addition of poly(A). The suitable fragments as judged by agarose gel electrophoresis were collected and used as templates for PCR amplification. The cDNA library of *R. labralis* was sequenced on the Illumina HiSeq™ 4000 platform under an effective concentration by Gene Denovo Biotechnology Co. (Guangzhou, China).

### De novo assembly, read mapping and bioinformatic analysis

The raw reads produced by the sequencing instrument were filtered to remove adaptors, low-quality sequences with unknown nucleotides N, and reads with more than 20% low quality bases (base quality < 10). The high quality clean reads were assembled into unigenes using the short reads assembling program Trinity (version2.0.6) [29]. The gene functions and classification were analysed based on searches against the following databases: the National Center for Biotechnology Information (NCBI) non-redundant nucleotide (Nr), the Swiss-Prot database, EuKaryotic Orthologous Groups (KOG), and the Kyoto Encyclopedia of Genes and Genomes (KEGG) database. Next, we used the Blast2GO program to obtain a GO annotation of the transcripts [30], and the GO function was classified by the WEGO software [31]. The coding sequences (CDSs) were predicted by using the best-matched fragments of the annotated unigenes that were obtained using BLASTx with a threshold E-value of 10^− 5^ against protein databases in Nr, Swiss-Prot, KEGG, and KOG. Proteins showing the highest scores in the BLASTx results were used to determine the CDSs of the unigenes. The CDSs of the remaining un-annotated unigenes were predicted using ESTScan.

### Differentially expressed genes (DEGs) and trend analysis

After the expression level of each gene was calculated and normalized by RPKM (reads per kb per million reads), differential expression analyses among the workers, IWs and NRs were conducted using edgeR [32, 33]. The false discovery rate (FDR) was used to determine the threshold for the *p* value following multiple tests, and the FDR ≤ 0.05 threshold and an absolute value of the log2Ratio ≥ 1 were used to judge the significance of the gene expression differences in the analysis. To analyse the expression profiles of workers, the IWs and the NRs based on the RPKM values, Short Time-series Expression Miner (STEM) software (http://www.cs.cmu.edu/~jernst/ stem) was used to display the trends in these three stages [34]. All genes were divided into eight modules according to the expression pattern, and the p value of each module was calculated using Permutation Test method. All DEGs were mapped to terms in the Kyoto Encyclopedia of Genes and Genomes (KEGG) database (http://www.genome.jp/kegg/pathway.html), and we looked for significantly enriched pathways in DEGs using the hypergeometric test. The pathways with Q ≤ 0.05 were defined as the significantly changed KEGG pathways.

### Quantitative real-time PCR (qRT-PCR)

To verify the quantification of gene expression levels in the transcriptome sequencing, qPCR was performed for five Ras signalling pathway-related genes and a catalase gene for the workers, IWs and NRs. The gene-specific primers were designed by Primer 5.0. The primer sequences of beta-actin were obtained from *R. flavipes* [35]. The beta-actin gene used in this study was well validated in *R. labralis* [36]. The quantitative reaction was performed on Life technologies/Vii7. The reaction mixture (20 μL) contained 2 × SYBR Premix Ex Taq TM II 10 μL, 0.8 μL each of the forward and reverse primers, and 2 μL of template cDNA. PCR amplification was performed under the following conditions: 95 °C for 10 s, followed by 40 cycles at 95 °C for 5 s and 60 °C for 30 s, and finally, at 95 °C for 15 s, 60 °C for 30 s and 95 °C for 15 s. Following amplification, a melting curve analysis was performed to detect a single gene-specific peak and to check the nonspecific amplification. The relative gene expression levels were calculated using the 2^–ΔΔCt^ method. All qRT-PCR experiments were repeated in three biological and three technical replications.

## Additional files

Additional file 1: Data statistics of clean data (PDF 187 kb)

Additional file 2: Unigene assembly results (PDF 188 kb)

Additional file 3: Unigene length distribution statistics (PDF 187 kb)

Additional file 4: Statistical results of the assembly unigene annotation. (PDF 131 kb)

Additional file 5: Species distribution of the BLASTX results. The species distribution of the unigene BLASTX results against the NCBI-Nr protein database. The different colours represent different species. (PDF 131 kb)

Additional file 6: Assembly unigene GO database functional classification (PDF 202 kb)

Additional file 7: Assembly unigene annotation KEGG pathway map (PDF 202 kb)

Additional file 8: The DEGs GO enrichment column of Profile 5 (PDF 207 kb)

Additional file 9: In profile5, 31 pathways were significantly related to IWs differentiation into NRs (Q-value < 0.05) (ZIP 215 kb)

Additional file 10: The signalling pathways in profile 5 showed that the Ras signalling pathway regulated and activated the downstream effector pathway MAPK and the calcium signalling pathway during IW development into NRs. (PDF 135 kb)

Additional file 11: Our KEGG pathway analysis showed that the CAT gene, as a downstream gene in longevity regulating pathway, directly cause longevity. The expression of CAT was suppressed by Ras-P13k-Akt-FOXO pathway in an uncomfortable environment. (PDF 129 kb)

## Data Availability

All data analyzed during this study are included in this article and its additional files. All raw sequence reads have been deposited in the NCBI SRA database and are accessible through SRA accession number SRP201410. The assembled gene sequences have been deposited in the NCBI TSA database under accession number GHNP00000000.

## Abbreviations

cDNA: Complementary De oxy ribonucleic acid
CDS: Protein coding regions
DEG: Differentially expressed genes
FDR: False discovery rate
GO: Gene Ontology
IW_1_: isolated late instar female workers for 1 week
IW_2_: isolated late instar female workers for 2 weeks,
IWs: isolated workers
KEGG: Kyoto encyclopaedia of gene and genome
KOG: Eukaryotic orthologues group of genes
mRNA: messenger ribonucleic acid
NRs: neotenic reproductives
qRT-PCR: Quantitative real time Polymerase chain reaction
RPKM: Reads per kilo base per million
