# Supplementary material for: Transcriptome changes reveal the genetic mechanisms of the reproductive plasticity of workers in lower termites
Source: BMC Genomics. 2019 Sep 9;20:702. doi: 10.1186/s12864-019-6037-y (PMC6734246; doi:10.1186/s12864-019-6037-y)
Supplement: Supplementary file 1 — Data statistics of clean data (PDF 187 kb) [file 12864_2019_6037_MOESM1_ESM.pdf]

Additional files 1 Data statistics of clean data

samples	Reads	Q20（%）	Q30（%）	GC（%）
Worker-1	42671226	96.72	91.17	44.52
Worker-2	52643176	96.61	90.96	44.76
Worker-3	49210596	96.65	91.02	44.91
IW-1	41811194	96.92	91.60	44.69
IW-2	60629726	96.88	91.51	44.38
IW-3	58099462	96.81	91.34	44.76
NR-1	52634082	98.42	95.30	44.33
NR-2	48203708	98.40	95.24	43.98
NR-3	54961688	98.32	95.06	44.13
