# Supplementary material for: Transcriptome changes reveal the genetic mechanisms of the reproductive plasticity of workers in lower termites
Source: BMC Genomics. 2019 Sep 9;20:702. doi: 10.1186/s12864-019-6037-y (PMC6734246; doi:10.1186/s12864-019-6037-y)
Supplement: Supplementary file 2 — Unigene assembly results (PDF 188 kb) [file 12864_2019_6037_MOESM2_ESM.pdf]

Additional files 2　Unigene assembly results

Assembly statistics	
Total numble of unigene	112,954
N50	1,536
GC（%）	41.35
Mean length of unigenes(bp)	848
Max length(bp)	38,952
Min length(bp)	201
