# Supplementary figures and images for: Transcriptome changes reveal the genetic mechanisms of the reproductive plasticity of workers in lower termites
Source: BMC Genomics. 2019 Sep 9;20:702. doi: 10.1186/s12864-019-6037-y (PMC6734246; doi:10.1186/s12864-019-6037-y)

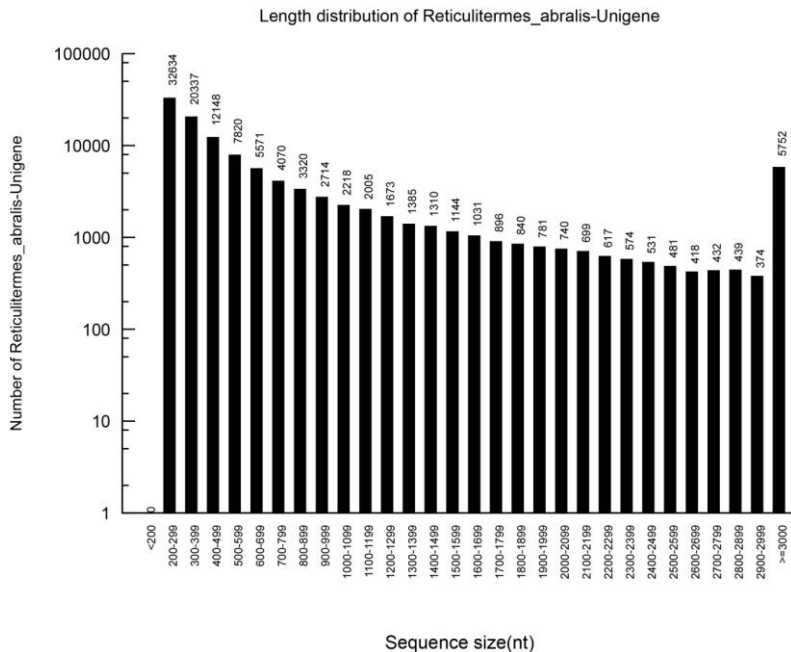

**Additional files 3 Unigene length distribution statistics**

Supplement: Supplementary file 3 — Unigene length distribution statistics (PDF 187 kb) [file 12864_2019_6037_MOESM3_ESM.pdf]

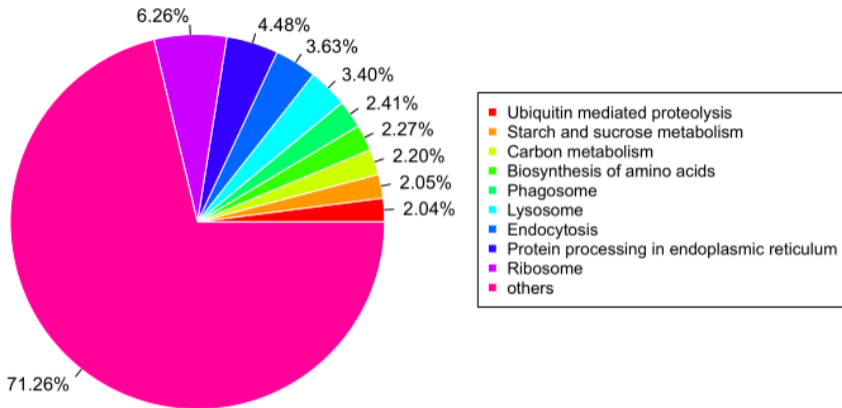

**Additional files 7** Assembly unigene annotation KEGG pathway map

Supplement: Supplementary file 7 — Assembly unigene annotation KEGG pathway map (PDF 202 kb) [file 12864_2019_6037_MOESM7_ESM.pdf]

Level2 GO terms of profile5

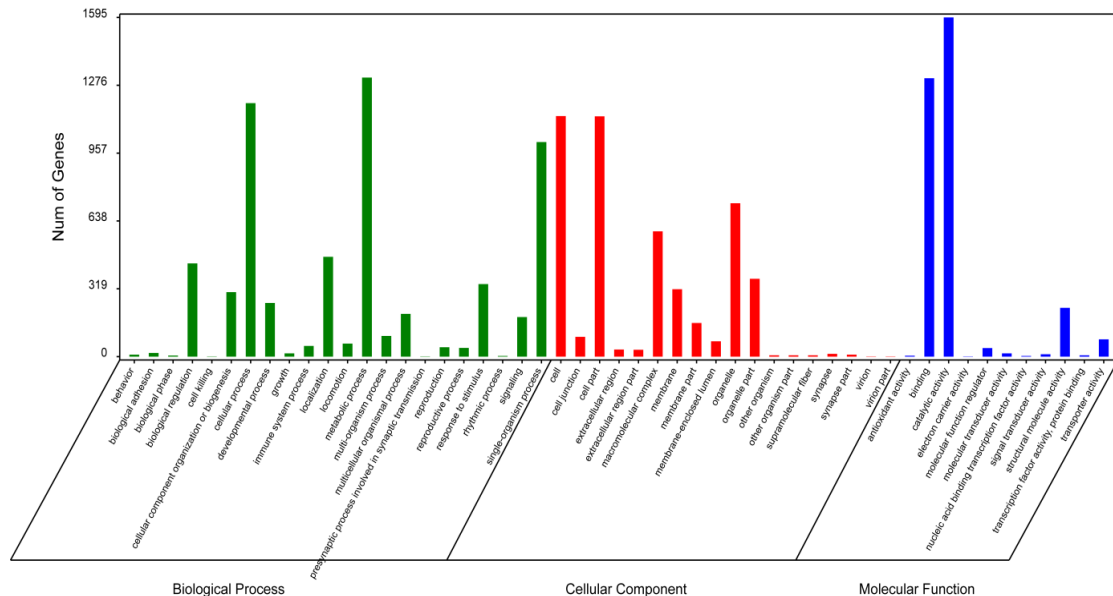

**Additional files 8** The DEGs GO enrichment column of Profile 5

Supplement: Supplementary file 8 — The DEGs GO enrichment column of Profile 5 (PDF 207 kb) [file 12864_2019_6037_MOESM8_ESM.pdf]
