# Supplementary material for: Transcriptome changes reveal the genetic mechanisms of the reproductive plasticity of workers in lower termites
Source: BMC Genomics. 2019 Sep 9;20:702. doi: 10.1186/s12864-019-6037-y (PMC6734246; doi:10.1186/s12864-019-6037-y)
Supplement: Supplementary file 4 — Statistical results of the assembly unigene annotation. (PDF 131 kb) [file 12864_2019_6037_MOESM4_ESM.pdf]

Additional files 4 Statistical results of the assembly unigene annotation

Annotated	Number of unigenes	Percentage (%)
Annotated in Nr	40,073	35.48
Annotated in SwissProt	29,540	26.15
Annotated in KOG	25,453	27.99
Annotated in KEGG	20,116	22.53
Annotated in all Databases	17,535	15.52
Annotated in at least one Database	40,972	36.27
without annotation genes	71,982	63.73
Total	112,954	100
