# Supplementary material for: Transcriptome changes reveal the genetic mechanisms of the reproductive plasticity of workers in lower termites
Source: BMC Genomics. 2019 Sep 9;20:702. doi: 10.1186/s12864-019-6037-y (PMC6734246; doi:10.1186/s12864-019-6037-y)
Supplement: Supplementary file 5 — Species distribution of the BLASTX results. The species distribution of the unigene BLASTX results against the NCBI-Nr protein database. The different colours represent different species. (PDF 131 kb) [file 12864_2019_6037_MOESM5_ESM.pdf]

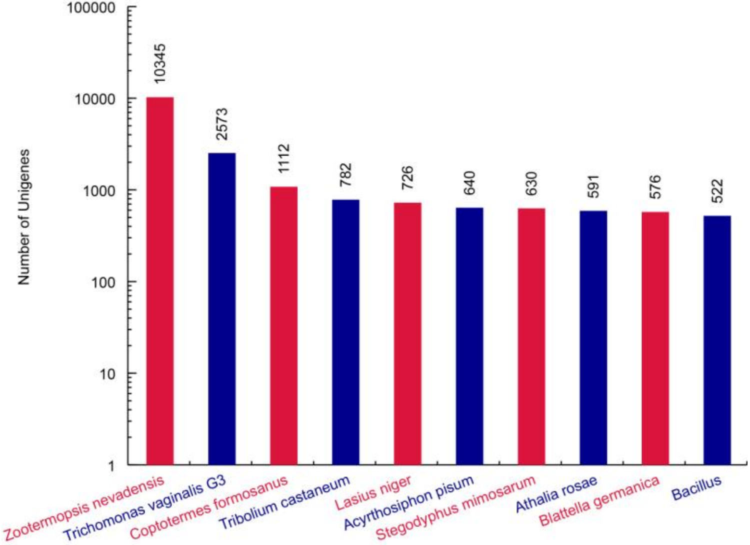

Additional files 5 Species distribution of the BLASTX results. The species distribution of the unigene BLASTX results against the NCBI-Nr protein database. The different colours represent different species.
