# Supplementary material for: Transcriptome changes reveal the genetic mechanisms of the reproductive plasticity of workers in lower termites
Source: BMC Genomics. 2019 Sep 9;20:702. doi: 10.1186/s12864-019-6037-y (PMC6734246; doi:10.1186/s12864-019-6037-y)
Supplement: Supplementary file 9 — In profile5, 31 pathways were significantly related to IWs differentiation into NRs (Q-value < 0.05) (ZIP 215 kb) [file 12864_2019_6037_MOESM9_ESM.zip › Additional files 9.pdf]

**Additional files 9** In profile5, 31 pathways were significantly related to IWs differentiation into NRs (Q-value <0.05)

| Pathway | DEGs genes with pathway annotation (2272) | All genes with pathway annotation (11902) | P value | Q value |
|---|---|---|---|---|
| 1. Ribosome | 391 | 1533 | 0.000000 | 0.000000 |
| 2. Protein processing in endoplasmic reticulum | 281 | 1098 | 0.000000 | 0.000002 |
| 3. Biosynthesis of amino acids | 158 | 556 | 0.000000 | 0.000002 |
| 4. NOD-like receptor signaling pathway | 27 | 52 | 0.000000 | 0.000005 |
| 5. Phosphatidylinositol signaling system* | 111 | 382 | 0.000001 | 0.000045 |
| 6. Cyanoamino acid metabolism | 63 | 193 | 0.000004 | 0.000150 |
| 7. Endocytosis | 219 | 888 | 0.000012 | 0.000329 |
| 8. Calcium signaling pathway* | 67 | 215 | 0.000013 | 0.000329 |
| 9. Starch and sucrose metabolism | 133 | 502 | 0.000021 | 0.000467 |
| 10. Progesterone-mediated oocyte maturation | 27 | 65 | 0.000024 | 0.000487 |
| 11. Amino sugar and nucleotide sugar metabolism | 101 | 366 | 0.000037 | 0.000673 |
| 12. Nitrogen metabolism | 25 | 62 | 0.000086 | 0.001445 |
| 13. Arginine biosynthesis | 36 | 104 | 0.000128 | 0.001839 |
| 14. GnRH signaling pathway | 36 | 104 | 0.000128 | 0.001839 |
| 15. ABC transporters | 52 | 169 | 0.000167 | 0.002078 |
| 16. Adrenergic signaling in cardiomyocytes | 49 | 157 | 0.000174 | 0.002078 |
| 17. Protein export | 48 | 153 | 0.000176 | 0.002078 |
| 18. PI3K-Akt signaling pathway* | 27 | 72 | 0.000196 | 0.002190 |
| 19. Ras signaling pathway* | 41 | 127 | 0.000259 | 0.002737 |
| 20. Neurotrophin signaling pathway | 21 | 55 | 0.000740 | 0.007441 |
| 21. Melanogenesis | 35 | 110 | 0.000948 | 0.009075 |
| 22. Fatty acid biosynthesis | 54 | 194 | 0.001810 | 0.016541 |
| 23. Vascular smooth muscle contraction | 41 | 139 | 0.001929 | 0.016858 |
| 24. ErbB signaling pathway* | 26 | 79 | 0.002408 | 0.020168 |
| 25. Estrogen signaling pathway | 30 | 96 | 0.002879 | 0.023144 |
