# Supplementary material for: Transcriptome changes reveal the genetic mechanisms of the reproductive plasticity of workers in lower termites
Source: BMC Genomics. 2019 Sep 9;20:702. doi: 10.1186/s12864-019-6037-y (PMC6734246; doi:10.1186/s12864-019-6037-y)
Supplement: Supplementary file 9 — In profile5, 31 pathways were significantly related to IWs differentiation into NRs (Q-value < 0.05) (ZIP 215 kb) [file 12864_2019_6037_MOESM9_ESM.zip › Additional files 9 continuiation.pdf]

26. Olfactory transduction	16	42	0.003154	0.024380
27. Alanine, aspartate and glutamate metabolism	42	148	0.003770	0.028066
28. Circadian entrainment	22	66	0.004221	0.030300
29. Oxytocin signaling pathway	28	91	0.004966	0.034422
30. T cell receptor signaling pathway	8	16	0.005177	0.034684
31. Long-term potentiation	23	73	0.007557	0.048996

* represents signal transduction pathways.
