# Supplementary material for: Transcriptome changes reveal the genetic mechanisms of the reproductive plasticity of workers in lower termites
Source: BMC Genomics. 2019 Sep 9;20:702. doi: 10.1186/s12864-019-6037-y (PMC6734246; doi:10.1186/s12864-019-6037-y)
Supplement: Supplementary file 10 — The signalling pathways in profile 5 showed that the Ras signalling pathway regulated and activated the downstream effector pathway MAPK and the calcium signalling pathway during IW development into NRs. (PDF 135 kb) [file 12864_2019_6037_MOESM10_ESM.pdf]

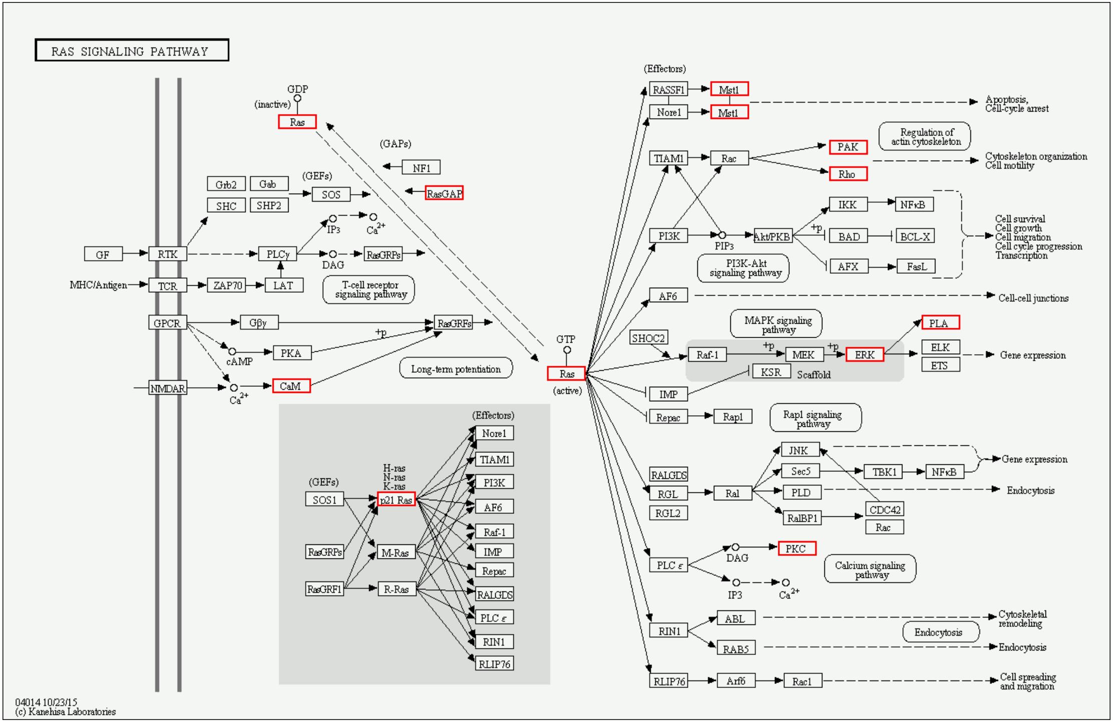

Additional files 10 The signalling pathways in profile 5 showed that the Ras signalling pathway regulated and activated the downstream effector pathway MAPK and the calcium signalling pathway during IW development into NRs.
