# Supplementary material for: Transcriptome changes reveal the genetic mechanisms of the reproductive plasticity of workers in lower termites
Source: BMC Genomics. 2019 Sep 9;20:702. doi: 10.1186/s12864-019-6037-y (PMC6734246; doi:10.1186/s12864-019-6037-y)
Supplement: Supplementary file 11 — Our KEGG pathway analysis showed that the CAT gene, as a downstream gene in longevity regulating pathway, directly cause longevity. The expression of CAT was suppressed by Ras-P13k-Akt-FOXO pathway in an uncomfortable environment. (PDF 129 kb) [file 12864_2019_6037_MOESM11_ESM.pdf]

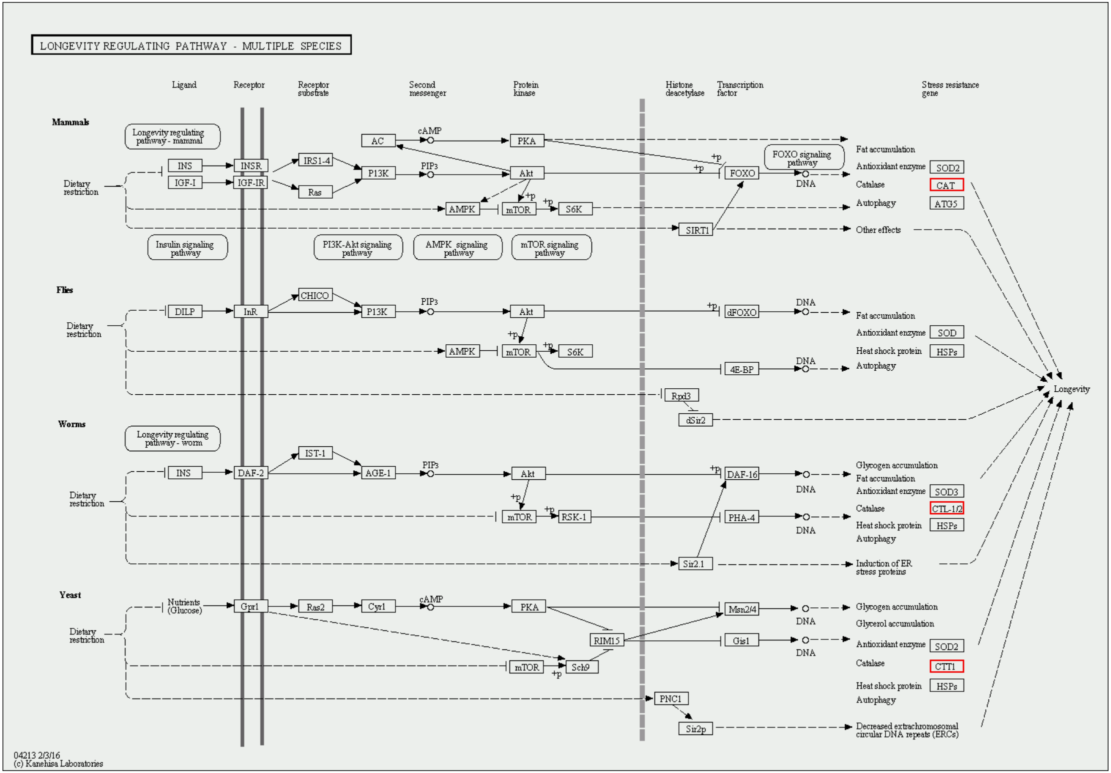

Additional files 11 Our KEGG pathway analysis showed that the CAT gene, as a downstream gene in longevity regulating pathway, directly cause longevity. The expression of CAT was suppressed by Ras-P13k-Akt-FOXO pathway in an uncomfortable environment.
