# Supplementary material for: Transcriptome changes reveal the genetic mechanisms of the reproductive plasticity of workers in lower termites
Source: BMC Genomics. 2019 Sep 9;20:702. doi: 10.1186/s12864-019-6037-y (PMC6734246; doi:10.1186/s12864-019-6037-y)
Supplement: Supplementary file 6 — Assembly unigene GO database functional classification (PDF 202 kb) [file 12864_2019_6037_MOESM6_ESM.pdf]

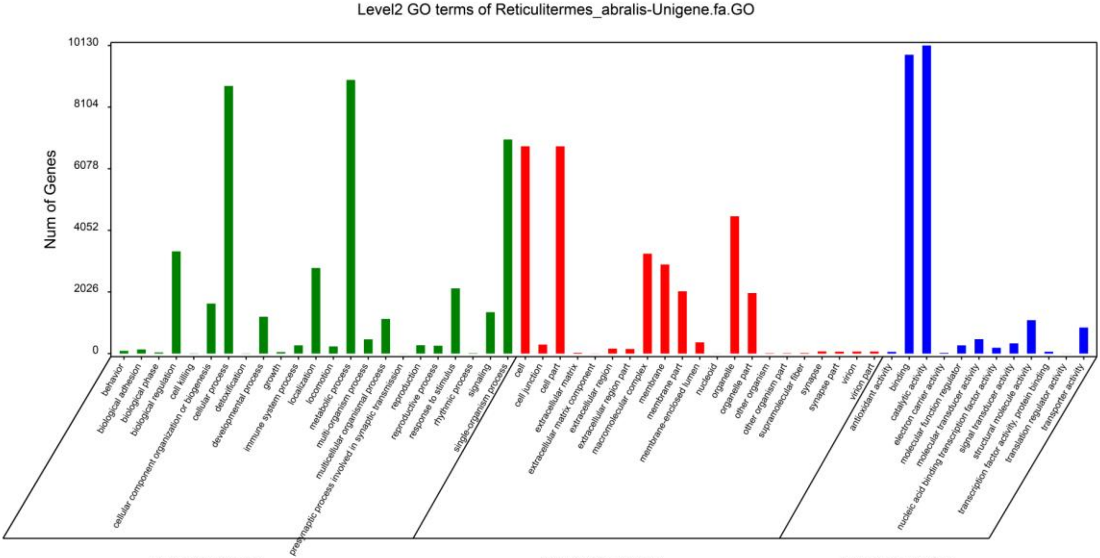

Additional files 6 Assembly unigene GO database functional classification
